# Matrine Mediates Inflammatory Response via Gut Microbiota in TNBS-Induced Murine Colitis

**DOI:** 10.3389/fphys.2019.00028

**Published:** 2019-02-08

**Authors:** Peiyuan Li, Jiajun Lei, Guangsheng Hu, Xuanmin Chen, Zhifeng Liu, Jing Yang

**Affiliations:** ^1^Department of Gastroenterology, The First Affiliated Hospital of University of South China, Hengyang, China; ^2^Xiangya School of Medicine, Central South University, Changsha, China; ^3^Department of Otorhinolaryngology, The First Affiliated Hospital of University of South China, Hengyang, China

**Keywords:** matrine, inflammation, gut microbiota, colitis, mouse

## Abstract

This study mainly investigated the effect of matrine on TNBS-induced intestinal inflammation in mice. TNBS treatment caused colonic injury and gut inflammation. Matrine (1, 5, and 10 mg/kg) treatment alleviated colonic injury and gut inflammation via reducing bleeding and diarrhea and downregulating cytokines expression (IL-1β and TNF-α). Meanwhile, serum immunoglobulin G (IgG) was markedly reduced in TNBS treated mice, while 5 and 10 mg/kg matrine alleviated IgG reduction. Fecal microbiota was tested using 16S sequencing and the results showed that TNBS caused gut microbiota dysbiosis, while matrine treatment markedly improved gut microbiota communities (i.e., Bacilli and Mollicutes). Functional analysis showed that cell motility, nucleotide metabolism, and replication and repair were markedly altered in the TNBS group, while matrine treatment significantly affected cell growth and death, membrane transport, nucleotide metabolism, and replication and repair. In conclusion, matrine may serve as a protective mechanism in TNBS-induced colonic inflammation and the beneficial effect may be associated with gut microbiota.

## Introduction

Inflammatory bowel diseases (IBD), an intestinal chronic inflammatory response or ulceration, is characterized by various pathologic symptoms, including bloody diarrhea, intestinal motility dysfunction, and intestinal shortening (Lee et al., 2014; Hirai and Matsui, 2015). The prevalence and incidence of IBD in China has markedly increased in recent years (Zhu et al., 2013). In the United States, about 1.0–1.5 million patients were estimated to suffer from IBD occurring between 2003 and 2004 (Kappelman et al., 2008). Although, the pathological mechanism of IBD is still unclear, compelling evidence suggests that inflammation and gut microbiota dysbiosis may serve as the major contributor in IBD (Ferguson et al., 2016). Thus, improving inflammatory status and gut microbiota communities may serve as a potential therapy for IBD patients.

Matrine, a kind of alkaloid substance, isolates from the roots of Sophora species in China. Compelling pieces of evidence have indicated that matrine exhibits various pharmacological activities, such as anti-inflammation, anti-oxidative stress, anti-infection, and cardiovascular protective effects (Liu et al., 2014; Cordero-Herrera et al., 2015; Yan et al., 2016). However, the merit of matrine on 2,4,6-trinitrobenzene sulfonic acid (TNBS)-induced murine colitis has not been fully studied. In this study, effects of matrine of intestinal inflammatory and gut microbiota in TNBS-induced murine colitis were mainly investigated.

## Materials and Methods

### Animal Model and Groups

This study was carried out in accordance with the recommendations of the Declaration of Helsinki. The protocol involving animal subjects was approved by the Animal Welfare Committee of the University of South China. Fifty female Balb/c mice (20.41 ± 1.68 g) were randomly divided into five groups with ten mice for each: normal control group (N group, *n* = 10), the TNBS group (TNBS group, *n* = 10), 1 mg/kg matrine plus TNBS (ML group), 5 mg/kg matrine plus TNBS (MM group), and 10 mg/kg matrine plus TNBS (MH group). Chronic colitis in mice was induced by weekly administration of increasing dosages of TNBS eight times (1.0–2.3 mg in 45% ethanol) according to previous report (Weiss et al., 2015; Levit et al., 2018). After 8 weeks, all mice were sacrificed for sample collection. Colonic length and weight were recorded.

### Clinical Evaluation of TNBS Colitis

Rectal bleeding and diarrhea of all mice in this study were recorded. Stool bloody level was determined by haemoccult kits (Beckman Coulter). Bloody stool was evaluated by the following scoring system: 0 means no blood in the stool; 2 means positive haemoccult in the stool; and 4 means gross bleeding in the stool. Diarrhea was evaluated by the following scoring system: 0 means well-formed pellets; 2 means pasty and semiformed stools; and 4 means liquid stools (Vlantis et al., 2015).

### Serum Immunoglobulins (Igs)

Blood samples were harvested by eye blooding and serum was separated by centrifugation (3,000 × g, 10 min, 4°C). Serum samples were stored at -80°C before Igs (IgA, IgG, and IgM) analysis by spectrophotometric kits (Nanjing Jiangcheng Biotechnology Institute, China).

### Real-Time PCR

Gut pro-inflammatory cytokines were determined to evaluate inflammation by real-time PCR. One piece of jejunum, ileum, and colon were harvested and stored at -80°C. Total RNA of these tissues was isolated using TRIZOL regent and reverse transcribed into the first strand (cDNA) with DNase I, oligo (dT)^20^ and Superscript II reverse transcriptase (Invitrogen, United States). The reverse transcription reaction was carried at 37°C for 15 min, 85°C 5 s. Primers in this study were designed with Primer 5.0 (Table 1). β-actin was selected as the house-keeping gene to normalize the expression of target genes. The PCR cycling used followed these conditions: 40 cycles at 94°C for 40 s, 60°C for 30 s, and 72°C for 35 s. The relative expression of target genes was normalized as a ratio to the expression of β-actin in the control group using the formula 2^-(ΔΔCt)^, where ΔΔCt = (Ct_Target_-Ct_β_–_actin_)_Treatment_-(Ct_Target_-Ct_β_–_actin_)_control_.

**Table 1:** PCR primer sequences: the forward primers (F) and the reverse primers (R) used in this study.

Gene	Nucleotide sequence of primers (5^′^–3^′^)	Accession
*β-Actin*	F:GTCCACCTTCCAGCAGATGT R:GAAAGGGTGTAAAACGCAGC	NM_008361.4
*IL-1β*	F:CTGTGACTCGTGGGATGATG R:GGGATTTTGTCGTTGCTTGT	NM_008361.4
*IL-10*	F: ACAGCCGGGAAGACAATAAC R: CAGCTGGTCCTTTGTTTGAAAG	NM_010548.2
*IL-17*	F:TACCTCAACCGTTCCACGTC R:TTTCCCTCCGCATTGACAC	NM_010552.3
*TNF-α*	F:AGGCACTCCCCCAAAAGAT R:TGAGGGTCTGGGCCATAGAA	NM_013693.3
*TLR4*	F: TTTGCTGGGGCTCATTCACT R: GACTCGGCACTTAGCACTGT	NM_021297.3
*Myd88*	F: CTCGCAGTTTGTTGGATGCC R: GGCCACCTGTAAAGGCTTCT	NM_010851.3


### Microbiota Sequencing

Total genome DNA from fecal samples was extracted for amplification using a specific primer (16S rRNA genes of distinct regions [Primer 16S V4, 515F:5^′^-GTGCCAGCMGCCGCGGTAA-3^′^ and 806R:5^′^-GGACTACHVGGGTWTCTAAT-3 ^′^)] (Burbach et al., 2017). Sequencing libraries were generated and analyzed according to our previous study. Observed-species, Chao1, Shannon, and Simpson are used to evaluate the complexity of species diversity. Phylogenetic Investigation of Communities by Reconstruction of Unobserved States (PICRUSt) was further used for genome prediction of microbial communities in this study (Douglas et al., 2018; Wilkinson et al., 2018).

### Statistical Analysis

All data in this study were analyzed using IBM SPSS 21.0 software. Comparisons between groups were analyzed by Tukey’s multiple comparison test after testing the homogeneity of variances via Levene’s test. Values in the same row with different superscripts (a, b, c) are significant (*P* < 0.05) (Liu et al., 2018a,b).

## Results

### Effects of Matrine on TNBS-Induced Colonic Injury

In this study, final body weight, colonic weight and length, rectal bleeding score, and diarrhea score were studied to evaluate clinical status TNBS-induced murine colitis. As shown in Table 2, TNBS markedly reduced body weight (27.72 ± 2.12 g) compared with the N group (33.47 ± 2.38 g) (*P* < 0.05). 5 and 10 mg/kg matrine significantly alleviated TNBS-induced growth suppression (*P* < 0.05). TNBS caused a marked colonic injury evidenced by the reduced colonic length and elevated colonic weight (*P* < 0.05). Although matrine failed to influence colonic length (*P* > 0.05), colonic weight was significant lower in the MM and MH groups than that in TNBS group (*P* < 0.05).

**Table 2:** Effects of matrine on clinical indexes. Data are presented as mean ± SEM.

Item	N group	TNBS	ML	MM	MH
FBW	33.47 ± 2.38^a^	27.72 ± 2.12^b^	28.57 ± 2.98^b^	29.83 ± 2.56^a^	30.11 ± 3.24^a^
CL	8.23 ± 1.20^a^	7.45 ± 0.98^b^	7.55 ± 1.11^b^	7.78 ± 0.87^ab^	7.92 ± 0.93^ab^
CW	148.62 ± 8.29^b^	196.27 ± 14.23^a^	199.36 ± 19.83^a^	188.29 ± 15.38^ab^	178.83 ± 11.62^ab^
RBS	0.00 ± 0.00^c^	4.12 ± 0.62^a^	4.04 ± 0.32^a^	3.79 ± 0.21^ab^	3.11 ± 0.19^b^
DS	0.00 ± 0.00^c^	3.48 ± 0.45^a^	3.11 ± 0.31^a^	3.02 ± 0.17^ab^	2.11 ± 0.21^b^


*The values having different superscript letters were significantly different (P < 0.05; n = 10). FBW: final body weight (g); CL: colonic length (cm); CW: colonic weight (mg); RBS: rectal bleeding score; DS: diarrhea score.*

Rectal bleeding and diarrhea score are two major clinical indexes and we found that TNBS treatment markedly increased rectal bleeding score and diarrhea score (*P* < 0.05), while 10 mg/kg matrine (MH) alleviated colonic bleeding and diarrhea (*P* < 0.05).

### Effects of Matrine on Serum Igs

As shown in Table 3, TNBS markedly reduced serum IgG level compared with the N group (*P* < 0.05), while 5 and 10 mg/kg matrine increased serum IgG level compared with the TNBS group (*P* < 0.05). In addition, dietary supplementation tended to enhance IgM production.

**Table 3:** Effects of matrine on serum immunoglobulins (g/l).

Item	N group	TNBS	ML	MM	MH
IgA	1.89 ± 0.18	2.33 ± 0.19	2.17 ± 0.22	2.22 ± 0.27	2.09 ± 0.16
IgG	9.36 ± 0.78^a^	7.23 ± 0.56^b^	7.97 ± 0.94^ab^	8.66 ± 0.87^a^	8.94 ± 0.67^a^
IgM	0.44 ± 0.04^ab^	0.37 ± 0.03^b^	0.42 ± 0.06^ab^	0.47 ± 0.05^ab^	0.55 ± 0.05^a^


*Data are presented as mean ± SEM. The values having different superscript letters were significantly different (P < 0.05; n = 10).*

### Effects of Matrine on Intestinal and Colonic Expression of Proinflammatory Cytokines

mRNA abundances of interleukin-1β (IL-1β), interleukin-10 (IL-10), interleukin -17 (IL-17), and tumor necrosis factor-α (TNF-α) were determined in the jejunum, ileum, and colon to evaluate gut inflammatory response (Table 4). In the jejunum, TNBS treatment upregulated IL-1β expression (*P* < 0.05), 5 and 10 mg/kg matrine alleviated TNBS-induced IL-1β over-expression (*P* < 0.05). Meanwhile, compared with the TNBS group, 10 mg/kg matrine markedly inhibited TNF-α expression (*P* < 0.05).

**Table 4:** Effects of matrine on intestinal and colonic expression of proinflammatory cytokines.

Item	N group	TNBS	ML	MM	MH
**Jejunum**					
IL-1β	1.00 ± 0.14^b^	1.61 ± 0.17^a^	1.42 ± 0.16^a^	1.26 ± 0.06^b^	1.15 ± 0.11^b^
IL-10	1.00 ± 0.12	1.07 ± 0.12	0.96 ± 0.05	0.89 ± 0.12	0.82 ± 0.22
IL-17	1.00 ± 0.19	1.27 ± 0.19	1.13 ± 0.18	1.21 ± 0.14	1.09 ± 0.16
TNF-α	1.00 ± 0.13^ab^	1.32 ± 0.23^a^	1.24 ± 0.16^ab^	1.03 ± 0.11^ab^	0.84 ± 0.09^b^
**Ileum**					
IL-1β	1.00 ± 0.07^b^	1.96 ± 0.15^a^	1.48 ± 0.24^b^	1.43 ± 0.10^b^	1.37 ± 0.23^b^
IL-10	1.00 ± 0.11	1.23 ± 0.19	1.12 ± 0.23	0.92 ± 0.07	1.22 ± 0.02
IL-17	1.00 ± 0.21	0.98 ± 0.11	1.07 ± 0.09	0.94 ± 0.05	1.63 ± 0.26
TNF-α	1.00 ± 0.17^b^	1.50 ± 0.27^a^	1.31 ± 0.05^ab^	1.28 ± 0.18^ab^	1.23 ± 0.11^ab^
**Colon**					
IL-1β	1.00 ± 0.20^b^	1.93 ± 0.09^a^	2.26 ± 0.31^a^	1.35 ± 0.48^b^	1.28 ± 0.07^b^
IL-10	1.00 ± 0.13^b^	1.45 ± 0.09^a^	1.34 ± 0.29^ab^	1.17 ± 0.38^ab^	1.21 ± 0.16^ab^
IL-17	1.00 ± 0.20	1.16 ± 0.12	1.15 ± 0.17	1.07 ± 0.72	1.10 ± 0.40
TNF-α	1.00 ± 0.12^b^	1.93 ± 0.21^a^	1.73 ± 0.16^a^	1.26 ± 0.24^b^	1.28 ± 0.10^b^


*Data are presented as mean ± SEM. The values having different superscript letters were significantly different (P < 0.05; n = 10).*

In the ileum, TNBS treatment markedly increased IL-1β and TNF-α mRNA abundances (*P* < 0.05), although matrine failed to mediate TNF-α expression in TNBS-induced murine colitis. Matrine (1, 5, and 10 mg/kg) significantly alleviated the overexpression of IL-1β (*P* < 0.05). In the colon, IL-1β, IL-10, and TNF-α were significantly upregulated in TNBS group compared with the N group (*P* < 0.05), and matrine (5 and 10 mg/kg) reduced IL-1β and TNF-α mRNA abundances (*P* < 0.05).

### Effects of Matrine on Intestinal and Colonic Expression of TLR4/Myd88

TNBS treatment markedly upregulated ileal Myd88 expression compared with the N group (*P* < 0.05) and matrine (5 and 10 mg/kg) inhibited ileal Myd88 expression (*P* < 0.05) (Table 5). Meanwhile, the mRNA abundances of TLR4 and Myd88 were significantly higher in the TNBS group than that in the N group in the colon (*P* < 0.05), while 10 mg/kg matrine alleviated colonic TLR4 activation (*P* < 0.05).

**Table 5:** Effects of matrine on intestinal and colonic expression of TLR4/Myd88.

Item	N group	TNBS	ML	MM	MH
**Jejunum**					
TLR4	1.00 ± 0.16	1.15 ± 0.17	1.14 ± 0.17	1.24 ± 0.15	1.13 ± 0.07
Myd88	1.00 ± 0.13	1.27 ± 0.18	1.29 ± 0.15	1.38 ± 0.16	1.34 ± 0.22
**Ileum**					
TLR4	1.00 ± 0.18	1.31 ± 0.06	1.30 ± 0.18	1.26 ± 0.13	1.34 ± 0.13
Myd88	1.00 ± 0.08^b^	1.71 ± 0.09^a^	1.43 ± 0.13^a^	1.14 ± 0.09^b^	1.23 ± 0.07^b^
**Colon**					
TLR4	1.00 ± 0.13^b^	1.52 ± 0.17^a^	1.55 ± 0.16^a^	1.34 ± 0.29^ab^	1.13 ± 0.22^b^
Myd88	1.00 ± 0.02^b^	1.72 ± 0.22^a^	1.42 ± 0.05^a^	1.64 ± 0.05^a^	1.23 ± 0.26^ab^


*Data are presented as mean ± SEM. The values having different superscript letters were significantly different (P < 0.05; n = 10).*

### Effects of Matrine on Gut Microbiota in TNBS-Induced Murine Colitis

16S rRNA sequencing yielded an average of 53,364 filtered partial sequences per sample with an average length of ∼300 bp. Alpha-diversity was tested by analyzing observed species, Chao1, Shannon, and Simpson (Table 6). Observed species, Chao1, and Simpson indexes were not altered in the TNBS and matrine groups (*P* > 0.05). Shannon value in the TNBS-treated mice was markedly lower than that in the normal group (*P* < 0.05), while matrine tended to increase the Shannon index (*P* > 0.05).

**Table 6:** Effects of matrine on gut microbiota alpha-diversity.

Item	N group	TNBS	MH
Observed species	609.33+76.19	587.38+61.25	594.82+53.46
Chao1	572.25+44.23	534.19+60.37	565.38+55.74
Shannon	4.62+0.32^a^	3.23+0.29^b^	3.75+0.43^ab^
Simpson	0.95+0.11	0.97+0.09	0.94+0.12


*Data are presented as mean ± SEM. The values having different superscript letters were significantly different (P < 0.05; n = 6).*

The overall microbial compositions in the TNBS and matrine groups were markedly changed at the class and family levels (Table 7). At class level, TNBS treatment markedly reduced the relative abundances of Bacilli and Mollicutes (*P* < 0.05), while matrine significantly restored the reduction of Bacilli and Mollicutes levels (*P* < 0.05). Meanwhile, matrine enhanced Betaproteobacteria and Bacteroidia levels compared with the N and TNBS groups (*P* < 0.05). At family level, Peptostreptococcaceae, Erysipelotrichaceae, Methylobacteriaceae, Sphingomonadaceae, and Lachnospiraceae were markedly reduced in response to TNBS-induced murine colitis, matrine treatment improved the relative abundances of Peptostreptococcaceae, Methylobacteriaceae, Sphingomonadaceae, and Lachnospiraceae (*P* < 0.05). Also, Bifidobacteriaceae was increased and Mycoplasmataceae was reduced in matrine-fed mice compared with the TNBS group (*P* < 0.05).

**Table 7:** List of significantly changed gut microbiota in response to TNBS and matrine treatments.

Item	N group	TNBS	MH
**Class level**			
Bacilli	0.3052 ± 0.0899^a^	0.0620 ± 0.0155^b^	0.2474 ± 0.0580^a^
Betaproteobacteria	0.0058 ± 0.0025^a^	0.0058 ± 0.0014^a^	0.0032 ± 0.0011^b^
Bacteroidia	0.0042 ± 0.0008^a^	0.0043 ± 0.0013^a^	0.0026 ± 0.0004^b^
Mollicutes	0.0028 ± 0.0006^a^	0.0018 ± 0.0006^b^	0.0025 ± 0.0010^a^
**Family level**			
Peptostreptococcaceae	0.0089 ± 0.0014^a^	0.0074 ± 0.0009^b^	0.0090 ± 0.0006^a^
Bifidobacteriaceae	0.0092 ± 0.0007^ab^	0.0087 ± 0.0006^b^	0.0099 ± 0.0014^a^
Erysipelotrichaceae	0.0048 ± 0.0006^a^	0.0041 ± 0.0009^b^	0.0041 ± 0.0005^b^
Methylobacteriaceae	0.0069 ± 0.0017^a^	0.0021 ± 0.0003^b^	0.0067 ± 0.0006^a^
Sphingomonadaceae	0.0087 ± 0.0026^a^	0.0019 ± 0.0005^b^	0.0066 ± 0.0009^a^
Mycoplasmataceae	0.0024 ± 0.0003^ab^	0.0041 ± 0.0024^a^	0.0015 ± 0.0005^b^
Lachnospiraceae	0.0028 ± 0.0003^a^	0.0019 ± 0.0003^b^	0.0027 ± 0.0005^a^


*Data are presented as mean ± SEM. The values having different superscript letters were significantly different (P < 0.05; n = 6).*

**FIGURE 1:**
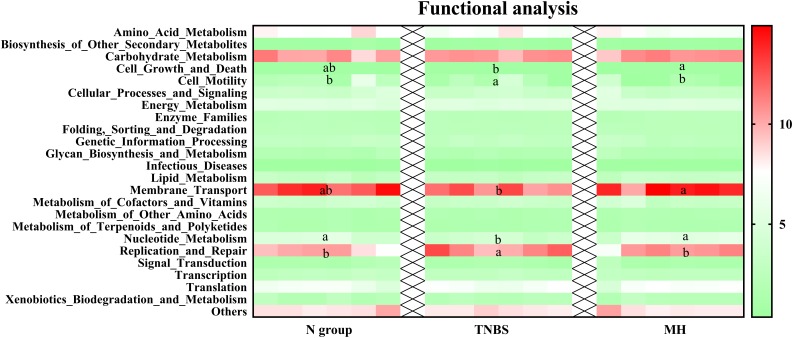
Genome prediction of microbial communities by PICRUSt analysis. Data are expressed as relative abundance of genes. The values having different superscript letters were significantly different (*P* < 0.05; *n* = 6).

PICRUSt was further used for genome prediction of microbial communities and the results showed that cell motility, nucleotide metabolism, and replication and repair were markedly altered in the TNBS group, while matrine treatment significantly affected cell growth and death, membrane transport, nucleotide metabolism, and replication and repair (Figure 1).

## Discussion

Previous reports indicated that mice receiving TNBS administration showed significantly increased clinical scores of rectal bleeding score and diarrhea score and body weight loss (Weiss et al., 2015; Zhang et al., 2015). In this study, we found that TNBS influenced final body weight, colonic weight and length, rectal bleeding score, and diarrhea score, suggesting a colonic colitis model. In addition, matrine exhibited a positive role in TNBS-induced colonic injury.

Igs are glycoproteins and one of the vital components of the immune system and previous reports suggested a beneficial role of Igs in inflammatory response (Brimelow et al., 2015; Elluru et al., 2015; Li et al., 2018). In this study, we found that IgG involves in colonic colitis as TNBS significantly inhibited serum IgG production. Meanwhile, matrine enhanced the serum IgG level, suggesting a protective role in TNBS-induced colonic injury. Matrine was demonstrated to be an immune enhancer via inducing T cell anergy in human Jurkat cells (Li et al., 2010). In this study, we firstly reported that matrine regulates serum IgG in TNBS-induced colonic injury.

IBD, including Crohn’s disease (CD) and ulcerative colitis (UC), are characterized by intestinal inflammatory response (Sands, 2015). In this study, TNBS caused intestinal inflammation and matrine exhibited an anti-inflammatory effect via mediating intestinal cytokines expression. In asthmatic mice, matrine attenuates allergic airway inflammation and eosinophil infiltration by suppressing eotaxin and Th2 cytokine production (Huang et al., 2014). In addition, matrine inhibits ovalbumin-induced airway hyperresponsiveness, inflammatory cell infiltration, and goblet cell differentiation via regulating Il-4, IL-13, and TNF-α expression (Sun D. et al., 2016).

NF-κB plays critical roles in development, survival, oxidative stress, inflammation and activation of B lymphocytes (Herder et al., 2015; D’Addio and Fiorina, 2016; Sasaki and Iwai, 2016; Jin et al., 2018). NF-κB was identified as one of the key regulators in the immunological setting. Its activation is markedly induced in IBD patients and promotes the expression of various pro-inflammatory genes (Atreya et al., 2008; Ruhl and Landrier, 2016). Thus, inhibition or inactivation of NF-κB serves as a potential therapy for IBD patients. In this study, we found that dietary matrine inhibited TLR4/Myd88 expression, the upstream signal of NF-κB. TLR4 is widely expressed in the intestine. Once activated by its ligands, TLR4 can activate NF-κB signaling pathway linked to the transcription of many proinflammatory genes (Tang et al., 2015). Compelling evidence has demonstrated that matrine regulates TLR4 expression (Liu et al., 2015; Sun N. et al., 2016). Furthermore, matrine can target NF-κB signal to regulate gene expression. For example, Lu et al. (2015) reported that matrine inhibits IL-1β-induced expression of matrix metalloproteinases by suppressing the activation of NF-κB in human chondrocytes *in vitro*. Similarly, matrine has been demonstrated to inactivate NF-κB signal in various cancer cells (Kim et al., 2013; Li et al., 2014).

Various previous studies have confirmed the role of gut microbiota in the pathophysiology of IBD (Peloquin and Nguyen, 2013; Gkouskou et al., 2014; Arora et al., 2018; Palamidi and Mountzouris, 2018; Roelofs et al., 2018; Zhang et al., 2018). The potential mechanism may be associated with the gut microbiota and host metabolism interaction as gut bacteria often target host metabolism, which further drive immune activation and chronic inflammation (Weingarden and Vaughn, 2017). Similar to previous studies, the current results showed that TNBS treatment caused microbiota dysbiosis by reducing alpha-diversity and the relative abundances of Bacilli and Mollicutes. Functional analysis showed that cell motility, nucleotide metabolism, and replication and repair were markedly altered in the TNBS group, while matrine treatment significantly affected cell growth and death, membrane transport, nucleotide metabolism, and replication and repair. The microbiota plays a fundamental role on the induction, training, and function of the host immune system and inflammatory response (Belkaid and Hand, 2014). Meanwhile, NF-κB activity has been reported to be affected by gut microbiota (Topol and Kamyshny, 2013). Thus, the gut microbiota might serve as a potential mechanism of the protective role of matrine in IBD models.

In conclusion, TNBS treatment induced colonic injury and inflammatory response in mice. Dietary matrine exhibited a protective role via enhancing serum IgG abundance and alleviating intestinal cytokines expression. The mechanism might be associated with gut microbiota as matrine improved gut microbiota communities in TNBS-induced murine colitis.

## Author Contributions

All authors listed have made a substantial, direct and intellectual contribution to the work, and approved it for publication.

## Conflict of Interest Statement

The authors declare that the research was conducted in the absence of any commercial or financial relationships that could be construed as a potential conflict of interest.
